# Pyodermatitis-pyostomatitis vegetans associated with autoimmune hepatitis: unreported co-existence 

**Published:** 2020

**Authors:** Samar Tharwat, Ehab E Eltoraby

**Affiliations:** *Rheumatology & Immunology Unit, Internal Medicine Department, Faculty of Medicine, Mansoura University, Egypt *

## Abstract

Pyodermatitis-pyostomatitis vegetans (PD-PSV) is a very rare inflammatory disease characterized by exudative pustular lesions of the skin and mucous membranes. The pathogenesis is unknown, but it may be related to immune or infectious processes. It is usually associated with inflammatory bowel disease (IBD). We describe a 32-year-old male with PD-PSV associated with manifestations suggestive of autoimmune hepatitis (AIH). To the best of our knowledge, this association has not been reported previously.

## Introduction

 Pyodermatitis-pyostomatitis vegetans (PD-PSV) is a rare chronic inflammatory disease presented with exudative plaques with elevated margins which affects the skin and mucous membranes (1). It is usually associated with inflammatory bowel disease (IBD) especially ulcerative colitis (2).The diagnosis is made based on clinical evaluation and characteristic histopathological changes as well as exclusion of malignancy and infection (3).

There is no standard therapy available for PD-PSV, with corticosteroids considered the first line therapy (4). The association with autoimmune hepatitis has not been previously reported. This paper aims to discuss an exuberant case of PD-PSV in a male patient with autoimmune hepatitis (AIH), emphasizing corticosteroid therapy management. 

## Case Report

A 32-year-old male admitted to our department with a chief complaint of two crusted, and ulcerated plaques over the face associated with pustular and vesicular painful lesions in his oral cavity. The lesions had begun insidiously 3 months earlier. The patient had also noticed yellowish discoloration in his eyes and skin 2 weeks before admission. The past medical and therapeutic history was unremarkable. 

Physical examination revealed diffusely jaundiced patient with skin, scleral icterus, a heart rate of 70 beats/min, respiratory rate of 18 breaths/min, temperature of 37.8° C, blood pressure of 120/80 mm Hg. The lungs were clear with no cardiac abnormalities, abdominal tenderness, or hepatosplenomegaly.

**Figure 1:**
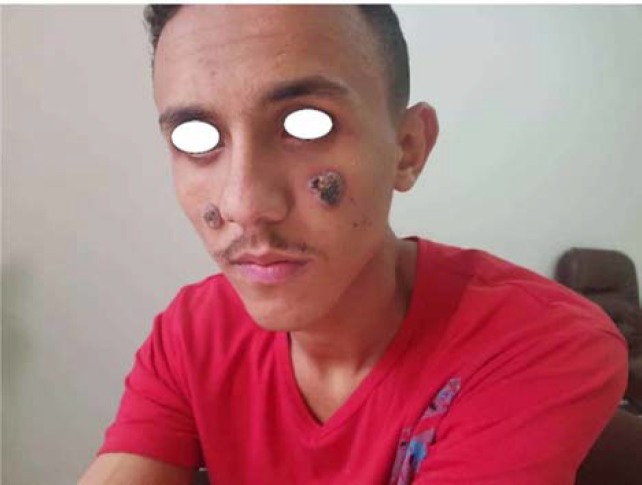
Plaques with raised borders and ulceration covered with crusts

**Figure 2:**
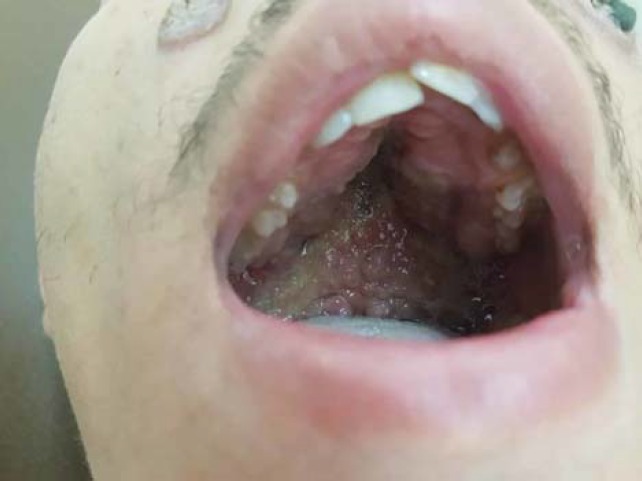
Multiple pustules on the erythematous base covered the oral mucosa

There were two ulcerated vegetating plaques with a papillomatous surface seen on the right and left checks (Figure 1). Oral examination revealed multiple yellowish small pustules with tender erythematous base covered buccal mucosa (Figure 2). The remainder of physical examination was unremarkable.

Histopathologic examination of a biopsy specimen taken from a plaque on his right check showed marked epidermal hyperplasia with crustation. The epidermis showed numerous dyskeratotic cells with collections of eosinophils and neutrophils. There was a dense dermal infiltrate of lymphocytes, eosinophils, and neutrophils with marked dermal edema. These findings were characteristic of pyodermatitis vegetans.

Laboratory investigations revealed red blood cell count 5.3× 106 /IU (4.20–5.40), hemoglobin 13.4 g/dL (12–16), hematocrit 41.5% (37.0–53.0), erythrocyte sedimentation rate 85 mm/h (0–20). The white blood cell count, platelets, electrolytes, glucose, BUN, creatinine were all within normal limits. Other results were; ALT 137 U/L (0-55), AST 81 U/L (0-34), serum albumin 3.9 gr/dl (3.5-5.2), total bilirubin 11.3 mg/dL; direct bilirubin, 8.3 mg/dL, INR 1. Chest X ray, abdominal USG revealed no pathology. He had blood cultures which yielded no pathological growth of any bacteria with several serological tests for toxoplasmosis, EBV, CMV, HBV, HCV, HAV, or HIV being all negative. Values of fasting glucose and lipid panel were normal. Ceruloplasmin, copper, and α-1-antitrypsin levels were normal. He had mild IgG elevation, together with negative ANA and positive ASMA in high titers. Liver biopsy revealed an inflammatory lymphocytic infiltrate in the portal areas and severe interface hepatitis suggestive of AIH. Taking into consideration the clinical presentation as well as the results of laboratory tests and histopathological examinations, the diagnosis was PD-PSV associated with AIH.

He was treated with prednisolone (1 mg/kg/day) for 4 weeks. The skin and mouth lesions significantly improved over the course of treatment, serum ALT, AST and IgG returned gradually to normal and total bilirubin significantly decreased. 

## Discussion

We presented a case of a young man who developed skin and mouth pustular lesions as a presenting manifestation of PD-PSV with associated manifestations of AIH. To the best of our knowledge, PD-PSV coexisting with AIH has not been reported previously. Treatment with systemic steroids resulted in resolution of skin and mucosal lesions, along with improvement of liver condition.

PD-PSV is a rare inflammatory disease affecting the skin and mucous membranes and frequently associated with IBD (5).The exact pathogenesis is unknown, but it seems to represent neutrophil-mediated altered mucocutaneous reactivity to an underlying systemic disease (6), or skin infections caused by pyogenic agents (7).

It is presented by vegetating plaques which may affect the scalp, face, axilla, and groin (8).Pyostomatitis vegetans is the oral equivalent of pyoderma vegetans on cutis (9). Histopathological changes include intra- or subepithelial abscesses containing eosinophils and neutrophils. There may also be mixed inflammatory cell dermal infiltrate, with hyperkeratosis, acanthosis, and reactive multinucleate keratinocytes (10).

The diagnosis is based mainly on clinical features, peripheral eosinophilia, negative culture of pus from lesions , histopathological features, and association with inflammatory bowel disease (11).

There have been multiple therapeutic agents used in the treatment of PD-PSV with topical or systemic corticosteroids as first-line agents (5). Dapsone, azathioprine, mycophenolate mofetil, cyclosporine, and infliximab, have all been used as second-line agents with varying success (12).

It has been described in patients with different disorders other than inflammatory bowel diseases e.g. severe psoriatic arthritis (13), myelodysplastic syndrome (14), infection with nocardia vinacea (15), staphylococcus aureus (16), lymphomas (17), HIV infection (18), alcoholism, nutritional deficit, or diabetes mellitus (19).

However, to the best of our knowledge, it has never been related to autoimmune hepatitis. It would be of great interest to understand the relationship between these mucocutaneous lesions and autoimmune hepatitis. Autoimmune hepatitis is known to be associated with several cutaneous manifestations (20). However, there is no published case describing PD-PSV in association with AIH. 

The pathogenesis of PD-PSV is still unknown; however, it could be related to underlying immune-mediated mechanism. AIH and PD-PSV may have a common pathogenic pathway. This case highlights the association between PD-PSV and AIH.

## Conflict of interests

The authors declare that they have no conflict of interest.
